# Bicuspid Aortic Valve: Role of Multiple Gene Variants in Influencing the Clinical Phenotype

**DOI:** 10.1155/2018/8386123

**Published:** 2018-09-05

**Authors:** Elena Sticchi, Rosina De Cario, Alberto Magi, Sabrina Giglio, Aldesia Provenzano, Stefano Nistri, Guglielmina Pepe, Betti Giusti

**Affiliations:** ^1^Department of Experimental and Clinical Medicine, Section of Critical Medical Care and Medical Specialities, University of Florence, Italy; ^2^Marfan Syndrome and Related Disorders Regional Referral Center, Careggi Hospital, Florence, Italy; ^3^Excellence Centre for Research, Transfer and High Education for the Development of De Novo Therapies (DENOTHE), University of Florence, Florence, Italy; ^4^Department of Biomedical Experimental and Clinical Sciences 'Mario Serio', University of Florence, Italy; ^5^Medical Genetic Unit, Meyer Children's University Hospital, Florence, Italy; ^6^Cardiology Service, CMSR Veneto Medica, Altavilla Vicentina, Italy

## Abstract

*Background. *Bicuspid aortic valve (BAV) is a common congenital heart defect with increased prevalence of aortic dilatation and dissection. BAV has an autosomal dominant pattern of inheritance with reduced penetrance and variable expressivity. BAV has been described as an isolated trait or associated with other clinical manifestations in syndromic conditions. Identification of a syndromic condition in a BAV patient is clinically relevant in order to personalize indication to aortic surgery. We aimed to point out how genetic diagnosis by next-generation sequencing (NGS) can improve management of a patient with complex BAV clinical picture.* Methods and Results. *We describe a 45-year-old Caucasian male with BAV, thoracic aortic root and ascending aorta dilatation, and connective features evocative but inconclusive for clinical diagnosis of Marfan syndrome (MFS). Targeted (91 genes) NGS was used. Proband genetic variants were investigated in first-degree relatives. Proband carried 5 rare variants in 4 genes:* FBN1*(p.Asn542Ser and p.Lys2460Arg),* NOTCH1*(p.Val1739Met),* LTBP1*(p.Arg1330Gln), and* TGFBR3*(p.Arg423Trp). The two FBN1 variants were inherited in cis by the mother, showing systemic features evocative of MFS, but with a milder phenotype than that observed in the proband. Careful clinical observation along with the presence of the* FBN1 *variants allowed diagnosis of MFS in the proband and in his mother.* NOTCH1 *variant was found in mother and brother, not exhibiting BAV, thus not definitely supporting/excluding association with BAV. Interestingly, the proband, his brother and father, all showing root dilatation, and his sister, with upper range aortic root dimension, were carriers of a* TGFBR3 *variant.* LTBP1* might also modulate the vascular phenotype.* Conclusions. *Our results underline the usefulness of NGS together with family evaluation in diagnosis of patients with monogenic traits and overlapping clinical manifestations due to contribution of the same genes and/or presence of comorbidities determined by different genes.

## 1. Background

Bicuspid aortic valve (BAV) disease is the most common congenital heart defect, as it affects 1.3% of the adult general population [1]. Aortic dilatation is a common feature of BAV with a prevalence ranging from 20 to 84% [1], due to differences in assessment techniques, study population, aortic size thresholds, and heterogeneous nature of the disease related to different genetic and nongenetic individual and population background. An increased risk of aortic dissection has been reported for BAV patients, thus raising concerns for the proper timing of aortic surgery in these patients [2–4].

Familial clustering has been reported, mostly with an autosomal pattern of inheritance with reduced penetrance and variable expressivity [5]. Actually, the heritability of BAV has been estimated to be 89%, thus suggesting the presence of a relevant genetic contribution [6].

Furthermore, BAV has been described as an isolated trait or associated with other clinical manifestations in syndromic conditions [familial thoracic aortic aneurysm and dissection, Marfan syndrome (MFS), Loeys-Dietz syndrome, Andersen syndrome, Turner syndrome, William Beuren syndrome, Bosley-Salih-Alorainy syndrome, and Athabascan Brainstem Dysgenesis syndrome] [5, 7, 8]. Identification of a syndromic condition in a BAV patient is clinically relevant in order to personalize indication to aortic surgery [9, 10].

Different genes with divergent inheritance pattern have been described to be associated with BAV [6].* NOTCH1 *(9q34.3, OMIM 190198), a transmembrane receptor involved in signaling pathways regulating cell fate and cardiovascular development processes, represents the main gene demonstrated to be associated with BAV in both familial and sporadic forms [7, 11, 12]. Mutations in* NOTCH1* signaling genes have been associated with a wide spectrum of congenital heart defects [13].

Shortly thereafter,* GATA5 *(20q13.33, OMIM 611496) has been also demonstrated to be involved in cardiac morphogenesis and aortic valve development [14] implicated in BAV pathogenesis [7, 15, 16]. The contribution of additional genes, such as* AXIN1, EGFR, ENG, NKX2-5, NOS3, PDIA2, TGFBR1, *and* TGFBR2*, has been also suggested in BAV disease [5, 7, 13, 17].

Previous data from our group evidenced the presence of genetic variants in* FBN1* gene, encoding for fibrillin1, a component of connective tissue, in 2 isolated BAV patients with aortic root dilatation and in 2 MFS patients with BAV [18, 19]. Actually, a higher prevalence of BAV among MFS patients with respect to the general population was demonstrated, thus possibly suggesting common underlying mechanisms responsible for the phenotypic expression of these clinical disorders. Fibrillin-1 deficiency could be responsible for matrix alterations, which contribute to aortopathy associated with BAV and MFS [20]. Similar connective tissue histopathological features in BAV and MFS have been reported by some authors; moreover, the aortic wall in both MFS and BAV patients is characterized by an increased metalloproteinase (MMP) activity and a decreased fibrillin-1 expression [20, 21].

Nevertheless, differences in the anatomic site of aortic wall vulnerability in both conditions, MFS and BAV, were evidenced: the maximal aortic dilatation is observed at the level of the proximal thoracic ascending aorta in BAV patients, while it is mainly referred to the aortic root in MFS patients [22]. Therefore, due to differences and similarities between BAV and MFS aortic wall pathology, the pathogenetic mechanisms underlying these conditions are still matter of debate.

We describe here a comprehensive target gene capture/NGS approach, analyzing 91 genes associated with BAV, syndromic and nonsyndromic aortopathy, vessels or valves growth, and remodeling processes, on a patient affected by normally functioning BAV, aortic root, and thoracic ascending aorta dilatation, with connective features evocative but inconclusive for a connective tissue disorder like MFS. We found rare nonsynonymous exonic variants, in large part predicted to be damaging by in silico analysis, that segregated with his “complex phenotype”, characterized by several clinical manifestations, ranging from vascular alterations (BAV and aortic root/ascending aorta dilatation) to systemic features suggestive of overlapping syndromic traits.

## 2. Methods

**Figure 1:**
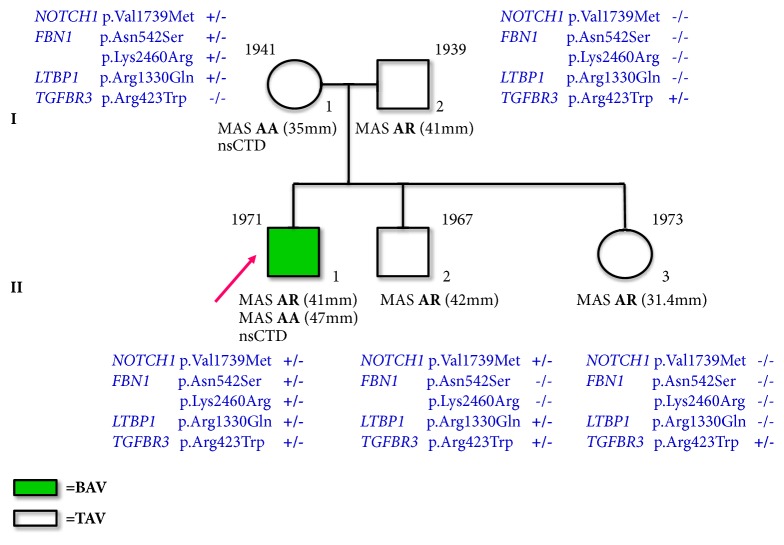
Pedigree of bicuspid aortic valve (BAV) family. BAV subjects are evidenced in green, whereas those showing tricuspid aortic valve (TAV) are not highlighted. An arrow indicates the proband case. Variants at* NOTCH1*,* FBN1*,* LTBP1*, and* TGFBR3* loci identified in BAV family members are shown. Birth years are reported at the upper left corner of symbols identifying subjects. MAS: maximal aortic size; AA: ascending aorta; AR: aortic root; nsCTD: nonspecific connective tissue disorders.

We describe a subject referred to the Center for Marfan Syndrome and Related Disorders (Careggi Hospital, Florence, Italy), affected by BAV, aortic root, and thoracic ascending aorta dilatation with a strong familiarity for thoracic aorta dilatation. His pedigree is reported in Figure 1. Our proband (II-1) was investigated by targeted NGS approach, and findings were validated through traditional Sanger sequencing. Thereafter, rare genetic variants identified in the proband have been investigated in first-degree family members [I-1 (mother), I-2 (father), II-2 (brother), and II-3 (sister)]. The experimental protocol was approved by the local ethical committee. All patients underwent genetic counseling and signed a written informed consent.

### 2.1. Echocardiographic Evaluation

All echocardiographic measurements had been made by a senior cardiologist (S.N.). BAV was diagnosed when only two cups were unequivocally identified in systole and diastole in the short axis view with a clear “fishmouth” appearance during systole as previously described [23, 24]. Aortic dimensions were assessed at end-diastole in the parasternal long-axis view at four levels by the leading edge method [23–25] and Z-scores were calculated according to age-adjusted nomograms for the aortic root [26]. Aortic or mitral regurgitation was evaluated and graded by multiple criteria combining color Doppler and continuous wave Doppler signals, and aortic valve stenosis was evaluated and graded by peak aortic valve velocity [23].

### 2.2. Genetic Analysis

Peripheral venous blood was collected in EDTA-coated vacutainer tubes and was stored at -20°C. Genomic DNA was extracted from blood samples using Tecan® Freedom EVO® liquid handling platform (Tecan Group Ltd, Switzerland) and GeneCatcher™ gDNA 0.3–1 mL Blood Kit (Thermo Fisher Scientific, USA). All procedures were performed following manufacturer protocols.

NGS was performed by 454 GS FLX Titanium technology (Roche, Basel, Switzerland). Using a targeted capture approach, we designed a panel of 91 genes regarded as relevant in BAV and syndromic and nonsyndromic aortopathy or involved in vessels or valves growth and remodeling processes by reviewing literature* (COL11A1, MTHFR, PLOD1, ADAMTSL4, MFAP2, PTGS2, SKI, TGFB2, CAPN2, AGT, MTR, TGFBR3, FGF8, RET, ACTA2, B3GAT3, LTBP3, EFEMP2/fbln4, LRP5, CCND1, LRP6, COL2A1, LRP1, DCN, LTBP2, TGFB3, FBLN5, ADAMTS17, CHST14, FBN1, SMAD3, MYH11, ABCC6, MAPK3, PDIA2, AXIN1, MMP2, CRYBA1, COL1A1, ACE, KCNJ2, EMILIN2, SMAD2, SMAD4, LTBP4, TGFB1, ADAMTS10, MMADHC, ACVR1, COL3A1, COL5A2, FN1, COL6A3, EMILIN1, LTBP1, JAG1, EMILIN3, MMP9, SLC2A10, GATA5, CBS, COL6A1, COL6A2, UFD1L, MAPK1, VHL, ZPLD1, MYLK, AGTR1, PDCD10, TGFBR2, FBN2, NKX2-5, B4GALT7, ADAMTS2, AGGF1, MTRR, NOS3, HOXA1, CCM2, KRIT1, COL1A2, TGFBR1, PTGS1, ENG, COL5A1, NOTCH1, GNAQ, AGTR2, FLNA, ELN: *870,000 bp). Genomic DNA libraries were prepared according to Roche sample preparation protocol (Rapid Library Preparation Method Manual, Roche Nimblegen Inc., Madison, WI). The customized capture array was designed to capture all coding exons, and flanking intron sequences of the 91 genes, according to the manufacturer's protocol (NimbleGen Arrays User's Guide: 454 Optimized Sequence Capture). Hybridization and washing procedures were performed according to Roche standard protocol. Sequencing run was then performed with the 454 Roche instrument. Sequencing runs have a mean coverage depth 45x.The standard flow files were analyzed with the GS Mapper software, version 2.6 (Roche Diagnostic, Basel, Switzerland).The gene variants calls were also evaluated by using an in-house pipeline developed by our bioinformaticians. Raw reads data were aligned to the human reference genome (hg19) with BWA version 0.7.12 [27]. Alignment and coverage statistics were generated with GATK version 3.3 [28], SAMTOOLS version 1.2 [29], and custom R scripts. Detection of single nucleotide variants and insertions/deletions was performed with the GATK Unified Genotyper by interrogating exons and intronic flanking regions (100bp) and taking into account strand-bias filtering and locus with more than 10X of sequencing coverage. Detected variants were annotated with Variant Effect Predictor version [30].

Sanger sequencing procedures were carried out in order to validate the identified genetic variants. The specific primer sets were designed using the Primer3 algorithm (http://frodo.wi.mit.edu/primer3). A bidirectional direct sequencing of PCR products was performed through ABI 3500Dx Sequencer (Applied Biosystems Foster City, CA, USA). Prediction of the effect of mutations was performed by Polyphen-2 (http://genetics.bwh.harvard.edu/pph2/), Sorting Tolerant from Intolerant (SIFT, http://sift.jcvi.org), FATHMM (http://fathmm.biocompute.org.uk/), and MutationTaster (http://www.mutationtaster.org/)* in silico* tools.

## 3. Results

The proband (II-1) is a 45-year-old Caucasian normotensive male (height 181 cm, weight 70 Kg). He presented with mild pectus excavatum and chest asymmetry, retrognathia, dolichocephaly, inguinal hernia, skin striae not associated with marked weight changes, and Beighton score 4/9. Echocardiography of the aorta documented the presence of a BAV with fusion of the right and left coronary cusps and a raphe and no calcification, dilatation of the aortic root (41mm; Z-score 2.56), and of ascending aorta (47 mm), and nonclassic mild mitral valve prolapse (i.e., without significant thickness of the valve leaflets) [31]. Magnetic resonance imaging (MRI) of the lumbosacral region did not show the presence of dural ectasia. No ocular clinical manifestations were observed in II-1. No family history of MFS or other connective tissue disorders and of aortic dissection or aortic/mitral valve replacement or aortic aneurysm was reported by the proband.

The clinical evaluation of the first-degree family members of II-1 was performed.

The mother (I-1) is a 75-year-old Caucasian normotensive female (height 164 cm, weight 54 Kg). She presents a positive (≥7) systemic feature score including myopia >3DO, mild pectus asymmetry and pectus carinatum deformity, mild thoracolumbar kyphosis, reduced elbow extension, and hindfoot deformity. Echocardiography of the aorta documented normal size of the aortic root diameters at the sinuses of valsalva within normal range (31 mm; Z-score -0.74), ascending aorta dilatation (35 mm), and mild mitral and tricuspid valve prolapse.

The father (I-2) is a 76-year-old Caucasian hypertensive male (height 177 cm, weight 89 Kg). He displays a systemic features' score of 4 for the presence of pectus carinatum deformity, pectus excavatum, and thoracolumbar kyphosis [8]. Echocardiography of the I-2 aorta documented the presence of aortic root at the sinuses of valsalva dilatation (41 mm; Z-score 1.19) with a normal size of the ascending aorta (32 mm).

The brother (II-2) of the proband is a 49-year-old normotensive Caucasian male (height 183 cm, weight 105 Kg) with pectus excavatum, pes planus, and striae distensae. He presented dilated aortic root (42 mm; Z-score 2.13) and normal size of the ascending aorta (30 mm).

The sister (II-3) of the proband is a 43-year-old normotensive Caucasian female (height 170 cm, weight 57 Kg) with severe scoliosis and osteoporosis. She presented normal aortic root (31 mm; Z-score 0.23) and ascending aorta (25 mm).

BAV was present only in the proband out of the 5 family members evaluated. In history taking of each member of the family there was no evidence of either occurrence of BAV or aortic surgery or sudden death.

**Figure 2:**
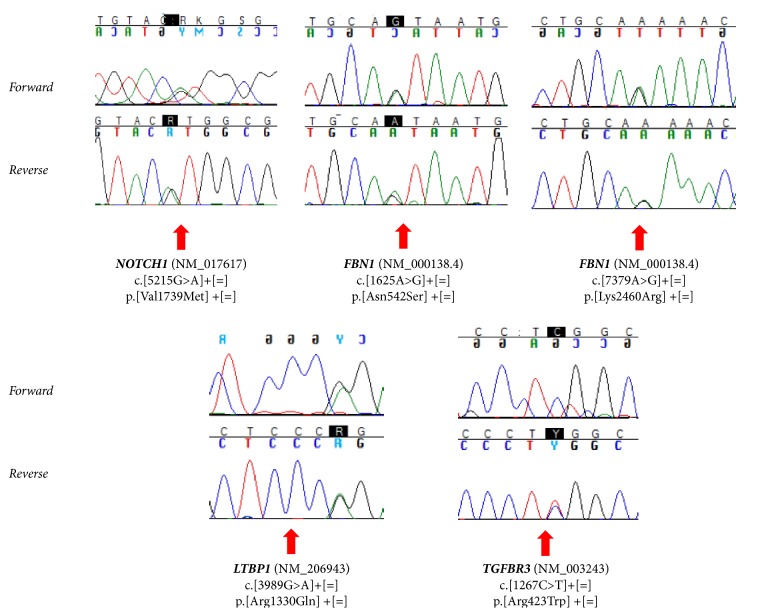
Electropherograms showing* NOTCH1*,* FBN1*,* LTBP1*, and* TGFBR3* gene mutations.

NGS targeted approach identified in the proband (II-1) 5 rare heterozygous variants [minor allele frequency (MAF)<0.01] (Table 1) in 4 different genes:* FBN1* (p.Asn542Ser and p.Lys2460Arg),* NOTCH1 *(p.Val1739Met),* LTBP1* (p.Arg1330Gln), and* TGFBR3* (p.Arg423Trp) (Figure 1). These variants were confirmed through Sanger technology (Figure 2).

Characteristics of identified variants and results from* in silico *prediction of the pathogenetic effect were reported in Table 1. Variants detected in the proband were also investigated in his first-degree relatives (Figure 1).* NOTCH1 *and* LTBP1 *mutations were present in proband's mother (I-1) and brother (II-2). Concerning* FBN1* gene, both p.Asn542Ser and p.Lys2460Arg variants were inherited* in cis *by the mother (I-1) and not found in any of other family members. Finally, the* TGFBR3* variant was transmitted by the father to the offspring (Figure 1).

In the proband, we also identified 139 common genetic variants (39 nonsynonymous) in the coding regions (data available if required).

## 4. Conclusions

In the present study, the designed targeted NGS allowed us to identify 5 rare, nonsynonymous exonic variants in a patient affected by complex phenotype with BAV, aortic root and thoracic ascending aorta dilatation, and connective features evocative for MFS. Traditional Sanger sequencing confirmed that these variants in* NOTCH1*,* FBN1*,* LTBP1*, and* TGFBR3 *genes were inherited, in various combinations, from the parents, one of which (mother) with a phenotype, upon closer observation, attributable to MFS. In fact, she presented the two different variants in* FBN1* gene and transmitted them* in cis* to the proband. According to the last revised Ghent criteria for MFS diagnosis, the identification of* FBN1* pathogenetic mutation in the proband, exhibiting aortic root dilatation (Z-score >2), allowed diagnosing MFS [8], and the identification of* FBN1* mutations in proband's mother also permitted diagnosing her as potential Marfan patient, even if with a less severe phenotype.

The two mutations in the* FBN1 *are c.1625 A>G (p.Asn542Ser) in the exon 13 and c.7379A>G (p.Lys2460Arg) in the exon 59. Both variants determine amino acid changes in calcium-binding epidermal growth factor-like domains (cbEGF4 and cbEGF38, respectively), known to mediate fibrillin assembly into microfibrils and in turn the biomechanical properties of the microfibrillar network [32]. Actually, both nucleotide substitutions do not affect highly conserved amino acids of the cbEGFs; nevertheless, some of* in silico* pathogenicity prediction tools showed a deleterious effect. Although c.7379A>G variant has been hypothesized to affect splicing process by generating a cryptic splice site,* in vitro* studies did not evidence its influence on mRNA processing [33]. This variant, reported in dbSNP database (rs144189837) with MAF≤0.0002 in Europeans, has been previously identified in patients with features of MFS [33, 34] and in thoracic* aortic* aneurysm (TAA) with a Marfan-like phenotype [35]. No information was available in literature or mutation databases for the c.1625 A>G variant. These two mutations were already identified together (not specified whether* in trans* or* in cis* due to lack of parents analysis) in our lab in further two patients: the first showing thoracic aortic dissection (TAD) type A and aortic valve substitution, mild myopia, striae distensae, altered lower/upper body segment ratio, and radicular cysts; the other, displaying a further* FBN1* mutation (c.6850C>A; exon 55), affected by severe classic MFS diagnosed on the presence of ectopia lentis, TAA and systemic features with score 10 including myopia, >3DO, pectus carinatum, scoliosis, wrist, and thumb signs, pes planus/hindfoot deformity.

According to international guidelines [10], the definition of MFS diagnosis in the proband determines the indication for an earlier aortic surgery. Moreover, in this patient, due to the presence of aortic root and ascending aorta dilatation, and genetic variants in* FBN1* and other potentially modifier genes, this issue seems of particular relevance. In fact, in this patient, further potentially pathogenetic variants for aortopathy were identified.

*NOTCH1* mutation is a nucleotide substitution from guanine to adenine at position 5215 in the coding gene sequence (c.5215G>A, exon 28), responsible for valine to methionine amino acid substitution at codon 1739 (p.Val1739Met), in the transmembrane domain of the encoded protein [11]. As cleavage of the intracellular domain, acting as a transcription factor able to modulate target genes expression [36], occurs at the transmembrane domain [37], it could be conceivable that modifications of this domain may result in an altered signal transduction. This variant, classified as damaging by most of* in silico* prediction tools (Table 1), has been described in dbSNP database (rs377294245), with a MAF of 0.00009 and has not been previously described in literature in BAV patients. Nonetheless, other* NOTCH1* mutations have been associated with BAV manifestations (http://www.hgmd.cf.ac.uk/ac/all.php). We have therefore identified this variant in proband's mother and brother, who, however, do not exhibit BAV. Nevertheless, due to the incomplete penetrance of BAV trait, the role of* NOTCH1 *as BAV causative gene cannot be longer excluded.

The c.3989G>A variant at* LTBP1* gene, also identified in the mother and in the brother, is predicted to be deleterious by Polyphen and Mutation Taster algorithm, and it might contribute to further modulate the phenotypic expression of the disease. LTBP1, a member of the LTBPs family, is known to have dual function: as structural component of the extracellular matrix and as modulator of TGF-beta availability essential for tissue formation and homeostasis [38, 39].

A mutation in the exon 8 of* TGFBR3* gene, encoding for the TGF-beta type III receptor often occurring as a coreceptor with other TGF-beta receptor superfamily members [40], was also found (c.1267C>T), with lying in the extracellular region of the protein and that cause amino acid substitution from arginine to tryptophan at codon 423 (p.Arg423Trp). Previous data from literature indicated the contribution of* TGFBR1* and* TGFBR2 *genes in thoracic aortic aneurysms and dissections [41, 42]. Only one study showed a* TGFBR2* mutation in a patient with BAV and TAA [43]. No mutations in* TGFBR1 *and* TGFBR2 *were identified in 11 unrelated Italian patients with familial BAV [13], as well as in previous further two studies on BAV patients [44, 45]. Interestingly, both father and brother, exhibiting aortic root dilatation, and sister of proband, in whom upper range of normal aortic root dimensions were observed, showed* TGFBR3* mutation, thus suggesting a cosegregation of* TGFBR3* variant with the presence of aortic root dilatation.

Beyond these rare genetic variants, other polymorphisms identified in the proband such as* MTHFR *p.Ala222Val (heterozygous) associated with severe cardiovascular manifestations in MFS patients or abdominal aortic aneurysm [46–48] or* NOS3 *p.Glu298Asp (heterozygous) associated with hypertension [49] and abdominal aortic aneurysm [50] could interact to modulate the clinical phenotype. Furthermore, a missense polymorphic variant (p.Ala84Thr), with a global MAF of 0.0274 (MAF 0.0109 in the European population), has been identified in* PLOD1* gene, whose expression/activity alterations have been suggested to be involved in impaired collagen cross-linking in BAV patients [51], so possibly contributing to modulating the clinical phenotype in addition to the* NOTCH1* mutation.

The presence of BAV in only one member of the family did not allow the definition of the contribution of rare or common genetic variants on aortic valve defect. In order to achieve a definite comprehension of larger BAV genetic bases, more informative families with several affected subjects are needed.

After a posttest genetic counseling, a clinical and echocardiographic follow-up to all the family members was recommended on a yearly basis for the proband, mother, father, and brother, biennial for the sister. Moreover, in absence of clinical news, an evaluation in the Marfan Center in Florence was also scheduled at every two years for all the family members. In particular, it was elucidated to the proband that, due to the MFS diagnosis, the threshold for aortic surgery was anticipated from that recommended for uncomplicated BAV (i.e., 55 mm) to the cut-off value recommended for MFS, i.e., 50 mm [9, 10].

On the bases of the current acknowledge [9, 10, 52, 53], a similar genetic approach should be taken into consideration, in BAV patients with thoracic aortic dilatation and further clinical manifestations suggestive of syndromic traits, and should be considered with more caution in other forms of BAV-associated aortic dilation patients.

In conclusion, our results underlined the complexity of clinical and genetic diagnosis also in traits considered monogenic, but in which clinical manifestations can overlap due to both contribution of the same genes to different phenotypes as well as presence of comorbidities determined by different genes. Moreover, this paper highlighted the usefulness of high-throughput sequencing technologies to improve diagnosis in a scenario in which an increasing number of genetic traits need to be considered polygenic/multifactorial. Target gene capture/deep sequencing approach can greatly improve the genetic diagnosis/management of patients with BAV. This study once more demonstrated the power of NGS in confirming and expanding clinical phenotypes/genotypes of the extremely heterogeneous conditions, assisting in genetic counseling and in carrier detection as well as providing therapeutic options.

## Figures and Tables

**Table 1:** Characteristics of rare variants identified in the proband case by NGS analysis.

**Gene**	**RefSeq**	**Exon**	**Variant description**	**dbSNP code** **MAF**	**In silico analysis**			
					**SIFT**	**POLYPHEN**	**FATHMM**	**MUTATION TASTER**
*NOTCH1*	NM_017617	28	c.[5215G>A]+[=] p.[Val1739Met]+[=]	rs377294245 MAF (ExAC) = 0.00009	Tolerated	Possibly Damaging	Damaging	Disease Causing
								
*FBN1*	NM_000138	13	c.[1625G>A]+[=] p.[Asn542Ser]+[=]		Tolerated	Benign	Damaging	Disease Causing
								
*FBN1*	NM_000138	59	c.[7379A>G]+[=] p.[Lys2460Arg]+[=]	rs144189837 MAF (ExAC) = 0.00007	Tolerated	Probably Damaging	Damaging	Disease Causing
								
*LTBP1*	NM_206963	26	c. [3989G>A]+[=] p.[Arg1330Gln]+[=]	rs141080282 MAF (EU)=0.005	Tolerated	Possibly Damaging	Tolerated	Disease Causing
								
*TGFBR3*	NM_003243	8	c.[1267C>T]+[=] p.[Arg423Trp]+[=]	rs766001542 MAF (ExAC) = 0.00003	Tolerated	Benign	Tolerated	Polymorphism

MAF: minor allele frequency; EU: European population; ExAC: ExAC Consortium population.

## Data Availability

All data supporting our findings are included in the article.

## Abbreviations

BAV: Bicuspid aortic valve
EGF: Epidermal growth factor
EU: European population
ExAC: ExAC Consortium population
MAF: Minor allele frequency
MFS: Marfan syndrome
MMP: Metalloproteinase
MRI: Magnetic resonance imaging
NGS: Next-generation sequencing
nsCTD: Nonspecific connective tissue disorders
OMIM: Online Mendelian inheritance in man
TAA: Thoracic aortic aneurysm
TAD: Thoracic aortic dissection
TAV: Tricuspid aortic valve
WES: Whole exome sequencing approach.
